# New Software for DQE Calculation in Digital Mammography Compliant with IEC 62220–1-2

**DOI:** 10.1007/s10278-021-00546-y

**Published:** 2022-09-14

**Authors:** Magdalena Dobrzyńska, Anna Wysocka-Rabin, Ewa Fabiszewska, Katarzyna Pasicz, Witold Skrzyński

**Affiliations:** 1 Particle Acceleration Physics and Technology Division, National Centre for Nuclear Research (NCNR), Otwock Swierk, Poland; 2 Medical Physics Department, Maria Sklodowska-Curie National Research Institute of Oncology (MSCNRIO), Warsaw, Poland

**Keywords:** Digital mammography, Modulation transfer function, Noise power spectra, Detective quantum efficiency, Software

## Abstract

Significant improvements in mammography systems have been achieved with the introduction of active matrix flat-panel digital detectors. The advent of this technology also makes it possible to implement computational methods for quantitative image analysis. This study describes new software created to perform detective quantum efficiency (DQE) calculations fully compliant with the IEC 62220–1-2 standard. Python-based software was developed that contains modules to calculate inverse conversion function, modulation transfer function (MTF), noise power spectrum (NPS), and DQE itself. A graphical user interface (GUI) and further add-ons make this software more user-friendly. Results are immediately displayed diagrammatically, and complete output data are exported to a .csv file. The code is available freely, as a compiled, executable file (.exe). The program was successfully tested using DICOM images obtained from mammography units from different manufacturers. This study also includes validation of the new software, based on comparisons of results obtained for the same set of data with two other, freely available programs.

## Introduction

The rapid transition from analog to digital mammography systems requires parallel changes in quality assurance methods. To optimize the benefits of digital imaging systems, improved testing protocols that apply computable, objective, and quantitative solutions should replace observer-dependent methods. However, introducing automated testing protocols in clinical practice should not make the procedure more complicated and time-consuming for clinical practitioners, so it is necessary to develop appropriate, user-friendly software.

The concept of detective quantum efficiency (DQE) as a standard for measuring radiographic image quality was introduced by Shaw in the early 1960s [1]. DQE was refined in a number of subsequent publications [2–6], and the present IEC standard [7] was published in 2007. Since that time, a few DQE programs have been developed [8–10]. All of these programs are based on the IEC standard, but they actually use a different DQE equation than the one published in the standard. Moreover, some of these codes are not completely compatible with some operating systems and some are not very convenient for a user. Our aim was to develop user-friendly DQE software that is fully compliant with the IEC standard [7].

In this work, we introduce new software for digital mammography DQE calculation, which is based on the exact equation and methods described in the IEC standard. It requires minimum user interactions, and we have observed that its step-by-step prompts are easy to follow, even for first-time users. We also present validation of our new software, in comparison to other freely available programs.

## Material and Methods

### Software

Our DQE software was developed in Python 3.7 language, which has a wide ranging library base. To make it more user friendly, the program employs a window-like GUI (graphical user interface) that was created using the Tkinter library. Because Python is an interpreted language, the code was compiled to the executable file (.exe) for Microsoft Windows, and it is available in English and Polish. Basic results are presented immediately on plots, and detailed data can be obtained from an additional .cvs file that is generated in results folder.

### DQE

As stated in IEC standard [7], the basic equation for frequency-depended DQE used in software is as follows:

1 $$\mathrm{DQE}\left(u,v\right)= {\mathrm{MTF}}^{2}\left(u,v\right)\frac{{W}_{\mathrm{in}}\left(u,v\right)}{{W}_{\mathrm{out}}\left(u,v\right)}$$

where.

MTF(*u,v*) is the pre-sampling modulation transfer function of the digital X-ray imaging device.

*W*_in_(*u,v*) is the input noise power spectrum of the radiation field at the detector surface, defined as follows:

2 $$W_{in}(u,v) = SNR_{in}^{2}\cdot K$$

where

SNR_in_^2^ is squared signal-to-noise ratio (Annex B of IEC standard [7])

*K *is measured air kerma at detector (KAD)

*W*_out_(*u,v*) is the noise power spectrum (NPS_out_) at the output of the digital X-ray imaging device.

**Fig. 1:**
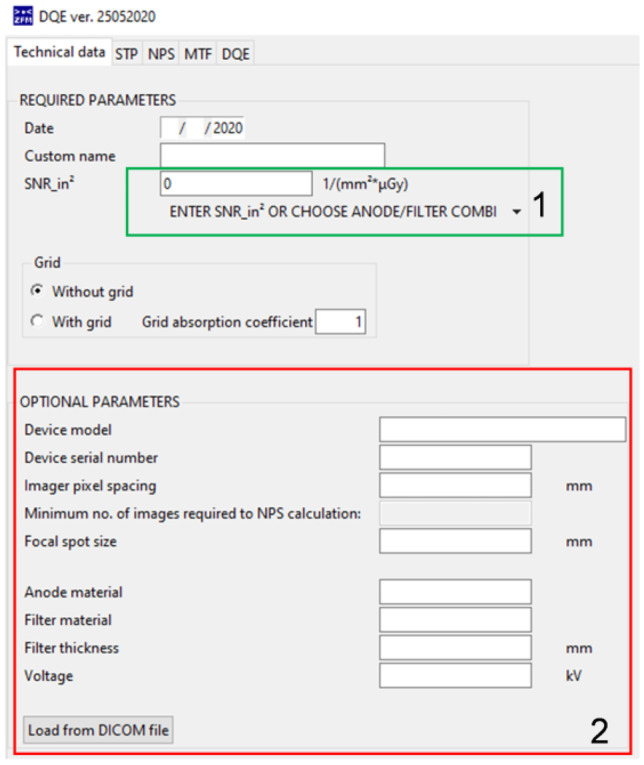
Example of first page of presented software

The new software allows the user to enter or choose SNR_in_^2^ value from a list (Fig. 1 (1) — in green), based on the anode/filter combination that was used. Built-in values come from the IEC recommendation [7] or are calculated using an online tool [11]. Additionally, the program folder has an additional text file that contains a list of SNR_in_^2^ loaded into program, so that the user can easily input additional values to the software.

### STP

IEC recommends that DICOM images used for NPS and MTF calculations should be normalized using an inverse conversion function. First, the system geometry should be described. However, as the IEC standard is based upon placement of the air kerma meter at the entrance to the image detector, a configuration which often is not possible under clinical conditions, measurements can be performed alternatively by placing the air kerma meter on top of the support table and then recalculating based on inverse square distance law. To simplify this step, the new software can do it automatically, based on distances (Fig. 1 (2), in red) entered by user (Fig. 1 (1), in green). This allows accurate calculation of the geometry correction coefficient.

**Fig. 2:**
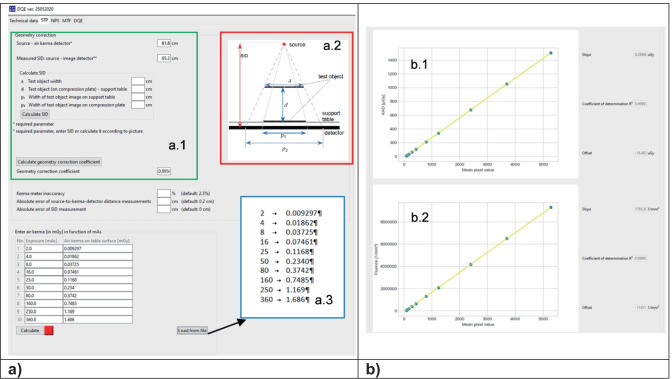
**a** Example of STP calculation window; **b** example of calculated functions

The program then asks the user to provide two sets of data: (1) measured air kerma in function of exposure (tube load — mAs) and (2) a set of DICOM images, made with the same tube loads as in (1). The data can be entered directly into the table or via text file (as shown in Fig. 2 (a.3) — with the additional window in blue).

From these data, the program calculates the dependency between mean pixel value and the air kerma and then fits these data to linear function (Fig. 2 (b.1)). The fit-results should fulfill the requirement of *R*^2^ ≥ 0.99 to be compatible with IEC standard. In the next step, this dependency is recalculated for photon fluence (instead of air kerma — Fig. 2 (b.2)) to establish the conversion function. Subsequently, the inverse conversion function is calculated. This function remains stored in the software memory until the program is closed so that the user does not have to remember about linearization of images to perform the next steps.

### NPS and NNPS

According to IEC standard [7], frequency-dependent noise power spectrum *W*_out_ is as follows:

3 $${W}_{\mathrm{out}}({u}_{n},{v}_{k})=\frac{\Delta x\Delta y}{M\times 256\times 256}\sum_{m=1}^{M}{\left|\sum_{i=1}^{256}\sum_{j=1}^{256}\left(I\left({x}_{i},{y}_{j}\right)-S\left({x}_{i},{y}_{j}\right)\right)\mathrm{exp}(-2\pi i\left({u}_{n}{x}_{i}+{v}_{k}{y}_{j}\right))\right|}^{2}$$

where

*x*, *y *is the distance between the pixel centers in horizontal and vertical direction, respectively

*M *is the total number of ROIs (region of interest)

*I*(x_*i*_,y_*j*_) is the linearized data

*S*(*x*_*i*_,*y*_*i*_) is the optionally fitted two-dimensional polynomial.

In this calculation, the user is asked to provide a set of flat panel images, with an irradiated area of approximately 100 mm × 100 mm. The method of making these images is described in detail in the IEC standard [7]. It is important that this calculation should be based on a sufficient number of images to ensure minimum 4 million pixels for analysis. If the user is not sure how many images should be made, the first page of the program provides a simple method to check that (Fig. 1 (1)— marked red).

**Fig. 3:**
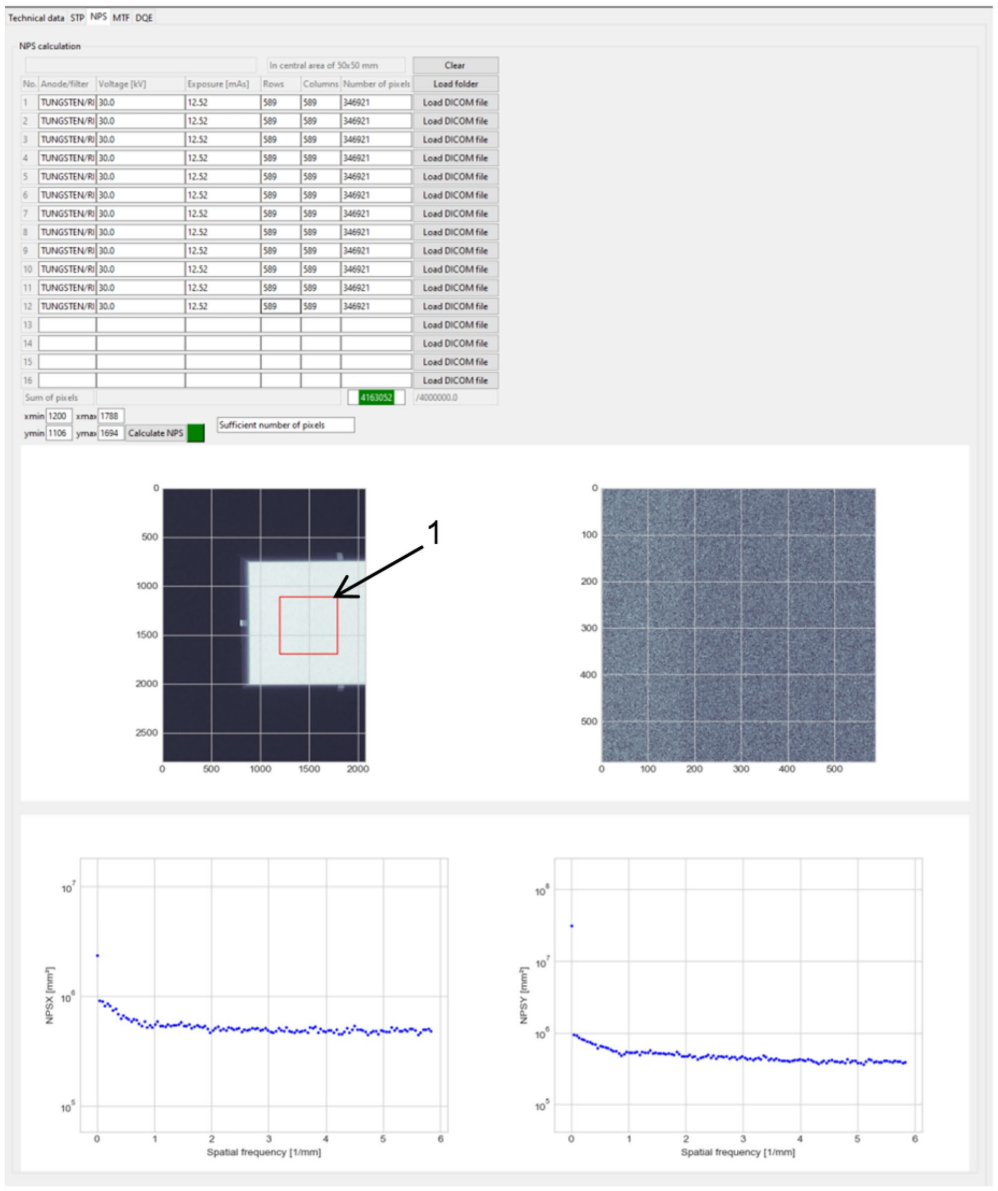
Example of NPS calculation window with loaded images and calculated results

NPS is calculated from an area of 50 mm × 50 mm (Fig. 3 (1), in red), divided into 256 × 256 pixel ROIs, with 128 pixel overlap. To achieve one-dimensional NPS from two-dimensional *W*_out_ result (Eq. (3)), data from 14 rows (or columns) around the axis are averaged (excluding axis itself). Additionally, normalized noise power spectrum (NNPS) is also calculated. The following equation was implemented to achieve NNPS [7]:

4 $$\mathrm{NNPS}=\frac{\mathrm{NPS}}{{\left(\mathrm{mean\ pixel\ value}\right)}^{2}}$$

### Trend Elimination

IEC describes *S*(*x*_*i*_,*y*_*i*_) two-dimensional polynomial function as an optional choice for trend removal purposes. The literature shows a further alternative method [12], which subtracts mean pixel value of flat field image. We checked the influence of the trend removal method on the final DQE and NPS result. Table 1 in the “Trend Elimination” section shows time of NPS calculation for one image and for set of 12 images (4 million pixels fulfilled).

**Table 1:** Durations of the calculations with different methods

Trend removal method	1 image	12 images
Without any method	< 2 s	< 5 s
Subtraction of mean pixel value	< 2 s	< 5 s
Subtraction of two-dimensional polynomial	ca. 2 min 40 s	ca. 19 min 30 s

### MTF

The modulation transfer function is calculated from images of the test plate described in IEC standard [7]. The user should provide two images: the first with the test edge placed perpendicular to the chest wall side of the detector and the second one, placed in parallel. Calculations are carried out separately for each image. Additionally, because calculations should be executed at the center of the edge, software can automatically find the proper position. Software also automatically calculates angle between edge and row (or column) of detector pixels.

**Fig. 4:**
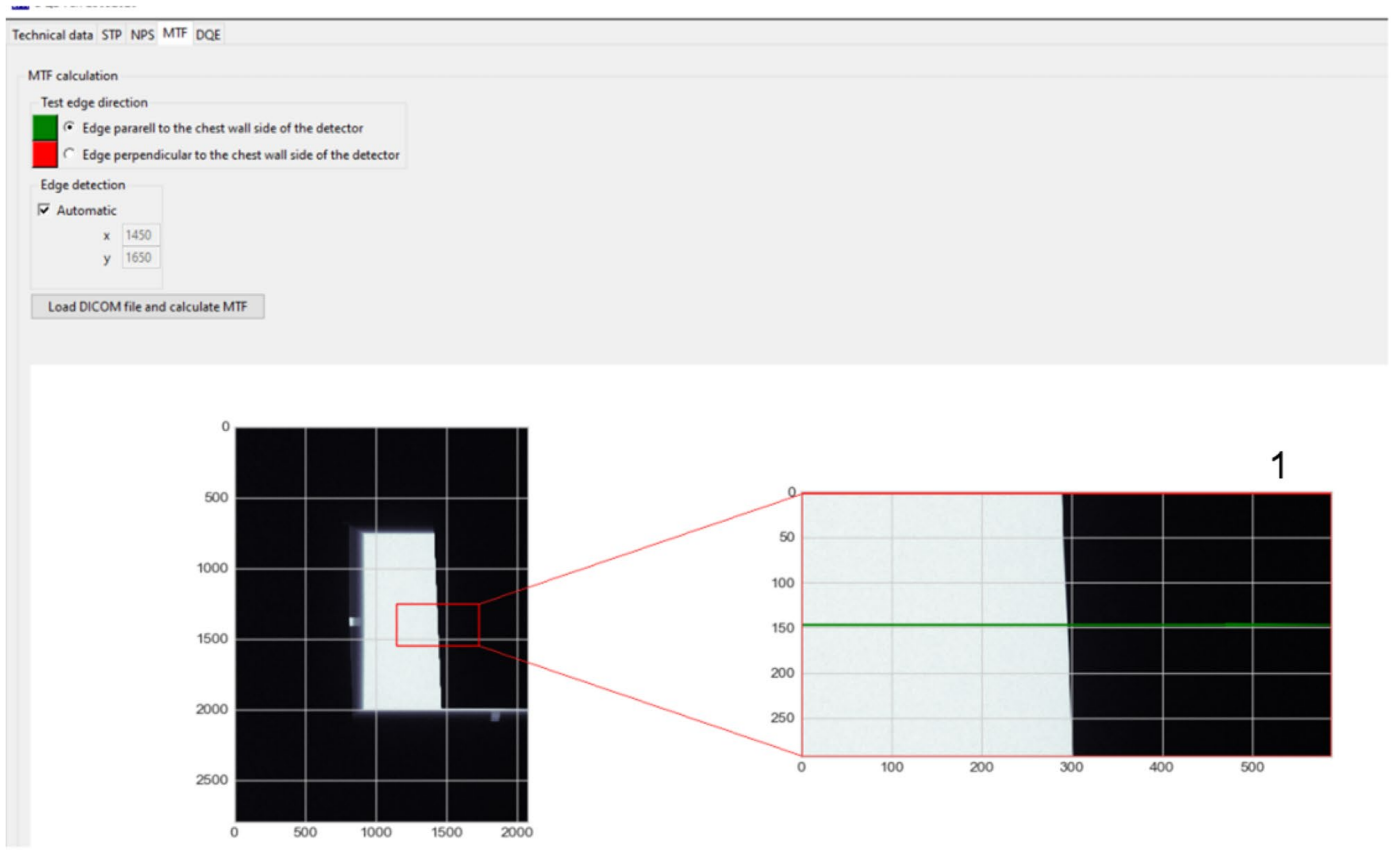
Example of MTF calculation window with loaded edge image

**Fig. 5:**
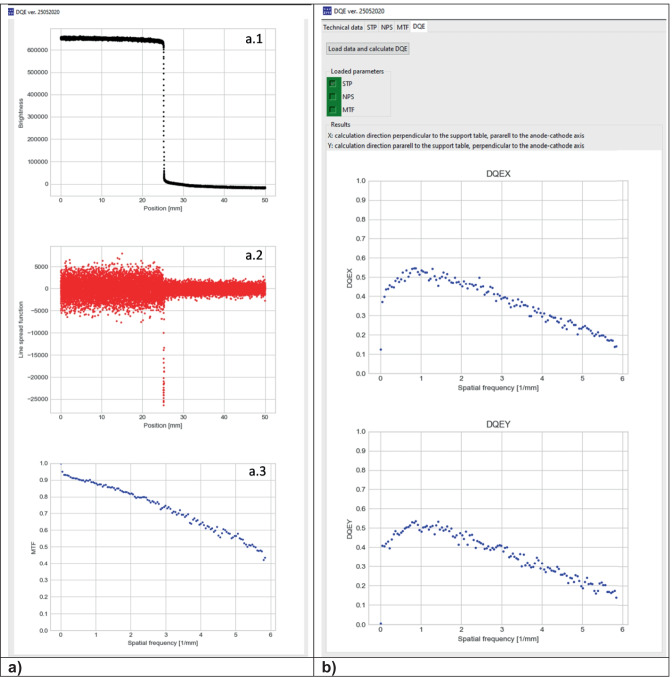
**a** Example of plotted ESF (**a.1**), LSF (**a.2**), and MTF (**a.3**) obtained by presented software. **b** Example of DQE calculation window with plotted DQEX and DQY results

The algorithm of MTF calculation is based on the IEC recommendation [7]. The oversampled edge spread function (ESF; Fig. 5 (a.1)) is calculated initially from the linearized edge image (with the edge at the center as shown). Based on edge angle (*α*), calculation window (Fig. 4 (1) — marked red) is divided into section with pixel lines number equal to 1/tg(*α*). The oversampled function from each section is determined using a sub-pixel method [13]. In the second step, the calculation of the line spread function (LSF is a derivative of ESF, with kernel [− 0.5, 0, 0.5]; Fig. 5 (a.2)) is performed. In the next step, a fast Fourier transform of LSF is calculated. To receive MTF value, it is necessary to normalize the FFT result to its value at zero-spatial frequency. The algorithm also uses a scaling factor of 1/cos*α* and finite-element differentiation correction [14], both recommended by IEC. Because ESF is oversampled, MTF values are re-binned to spatial frequency the same as NPS and limited by Nyquist value (Fig. 5 (a.3)).

### Uncertainty

Relative uncertainty reported in results (as u_DQEX and u_DQEY) was calculated using the Eq. (1) based on the instructions of GUM [15] and can be stated as follows:

5 $${u}_{\mathrm{DQE}}=\sqrt{{\left(2{u}_{\mathrm{MTF}}\right)}^{2}+{\left({u}_{{W}_{\mathrm{out}}}\right)}^{2}+{\left({u}_{\mathrm{NPS}}\right)}^{2}}$$

Uncertainty of MTF (*u*_MTF_) depends on the angle of the test plate, which causes a different number of lines in window used for calculation [16].

Uncertainty of NPS can be calculated [17] as follows:

6 $${u}_{\mathrm{NPS}}=\frac{1}{\sqrt{M \times row\times bin}}$$

where

*M *is the total number of ROIs

*row* is the number of rows used to calculate one-dimensional NPS (equal to 14 according to IEC standard [7])

*bin* is the multiplication of resolution and interval of NPS spatial frequency.

Uncertainty of *W*_in_ depends on the accuracy of the air kerma detector device and error of measurement or calculation of distances: source to image detector and source to air kerma detector. These two parameters should be entered by the user in the space provided on the STP card.

### DQE Calculation

After completing the steps described above, the user need only to click the “Load data and calculate DQE” button. All previously received results will be loaded, and DQE charts will be shown in the window. And additional .cvs file with detailed data will be generated and saved in the result folder in software's working directory. This file contains the following: NPS, NNPS, MTF, and DQE for two directions (*X* and *Y*) in function of spatial frequency resulting from FFT as well as DQE in two directions binned into multiplicity of 0.5 lp/mm. The file also contains basic information about analyzed data set: tube voltage, mAs, KAD, and calculated uncertainties.

### Measurements for Validation

During the validation process, the new software was successfully tested using data from three mammography systems at the Maria Sklodowska-Curie National Research Institute of Oncology (MSCNRIO) in Warsaw: Siemens Mammomat Inspiration, Hologic Selenia, and GE Senographe Pristina. For each system, the data sets contained images acquired for different anode/filter combinations, tube voltages, and mAs values. The same image sets were processed using MIQaELa [8], COQ plugin for ImageJ [9], and software described in this paper. For the purpose of clarity, only measurements from Siemens system are presented in this paper.

## Results and Discussion

### Trend Removal

Trend removal tests were executed on a computer with 4-core 1.60 GHz CPU, 16 GB RAM, and 64-bit Windows 10.

**Fig. 6:**
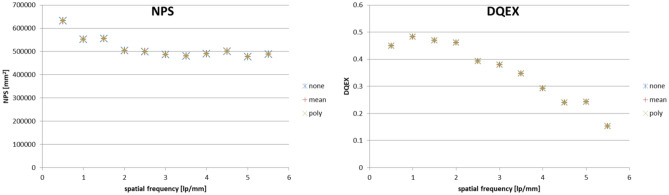
Comparison of NPS and DQE results for different trend removal methods; example for W/Rh, 30 kV, 12.5 mAs

Figure 6 presents charts of NPS and DQE obtained for each trend removal method (example for W/Rh, 30 kV, 12.5 mAs).

Calculated differences in values of NPS and DQE were insignificant for all trend removal methods, with maximum difference of 0.002% for both NPS and DQE. Based on the uncertainty section above, calculated uncertainty was 0.7% and 9.7% for NPS and DQE, respectively. The mean pixel value method was chosen as a compromise between including trend removal and calculation time. This solution covers possible cases when the data trend is stronger than that was observed in the images obtained.

### Validation

Validation of the new software described in this paper was performed for images obtained under the exposure conditions described in Table 2.

**Table 2:** Condition of exposure and calculation used in validation

Anode/filter combination	W/Rh
Tube voltage	30 kV
Tube load	12.5 mAs
SNR_in_^2^	6179 1/mm^2^µGy

**Fig. 7:**
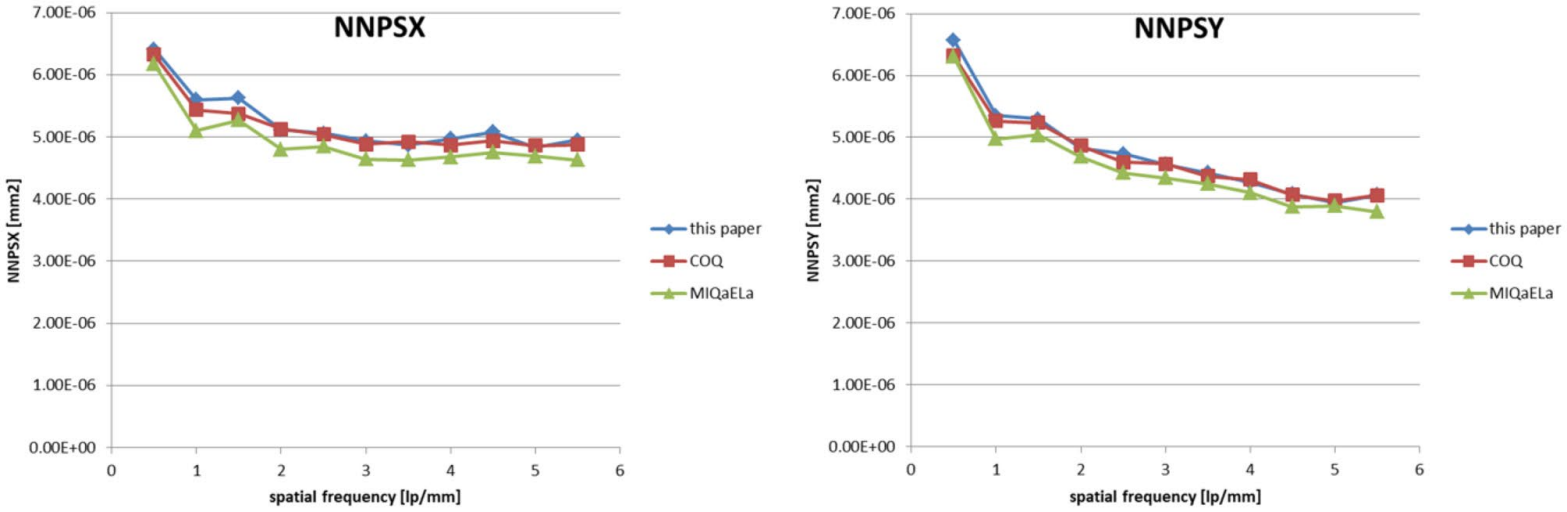
Comparison of calculated NNPS in *x* and *y* directions

**Fig. 8:**
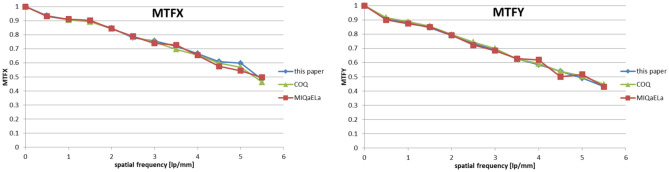
Comparison of calculated MTF in *x* and *y* directions

**Fig. 9:**
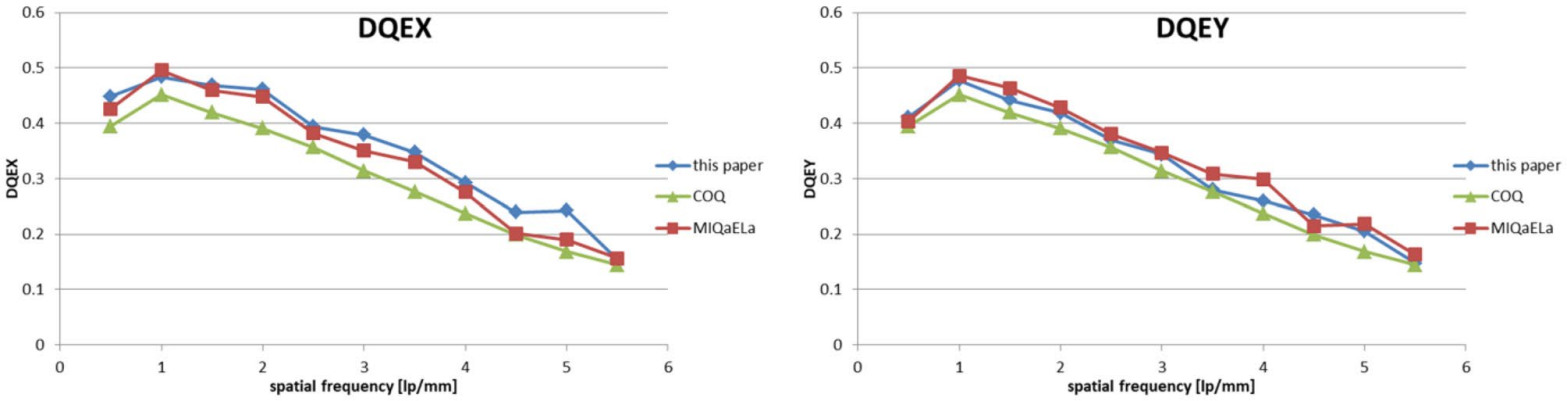
Comparison of calculated DQE in x and y direction

Figures 7–9 present results of NNPS, MTF, and DQE calculations performed with MIQaELa [8], COQ plugin for ImageJ [9] software, and the new software described in this paper.

Values of NNPS (Fig. 7) differ slightly when different programs are used. Our program agrees well with COQ. Values from MIQaELa are much lower; however, the authors of this program implemented different NNPS calculation methods (NPS is divided by squared kerma value instead of mean pixel value). Mean relative differences between results are 1% and 5.1%, with maximum difference 4.5% and 8.9% relative to COQ and MIQaELa, respectively. Calculated NPS uncertainty is 0.7%. It should be stated that MIQaELa and COQ use different DQE equations in which NNPS is a base value. In our software, NNPS is calculated only for user convenience and the base value is NPS as stated in 4 (Eq. (1)).

MTF (Fig. 8) shows a good agreement between values received with different programs. Mean relative differences between results are 1.4% and 0.4%, with maximum difference of 4.8% and 8.9% relative to COQ and MIQaELa, respectively. The calculated MTF uncertainty is 2.1%. Differences may result from different binning methods or location of the calculation window. The program presented in this paper allows for the possibility of finding the edge center. The other two programs require the user to determine of the area of calculation.

DQE results (Fig. 9) show a strong agreement with MIQaELa software. However, the comparison of DQE with data calculated using COQ is difficult, because this software presents only one DQE curve. The authors do not clarify to which calculation axis it refers, or what additional processing methods were used to obtain it. Mean relative differences between DQE results are 11.1% and 5% relative to COQ and MIQaELa, respectively. The calculated DQE uncertainty is 9.4%. Results from COQ are also visibly smoother, because for DQE, the authors used a 5th degree polynomial for data fitting. For our software, we did not decide to use this method, leaving the decision of choosing the method of presentation to the user. Our experience with DQE binning and fitting shows its strong sensitivity to the processing method. Differences in results among software can be caused by those specific methods applied. Additionally, investigation of spatial frequency alone suggests that the algorithm of a fast Fourier transform can slightly differ between programming languages (MATLAB for MIQaELa, Java for COQ and Python [NumPy library] for our software).

## Conclusion

As a result, we developed simple software fully consistent with IEC recommendations [7]. Its step-by-step structure and graphic user interface make it easy to use even for novices. It has been successfully tested on various sets of data. In this paper, a shortened method of data and image acquisition was also presented. Results show good agreement with data obtained using other software, with differences mainly caused by the equations used or the calculation routines employed.

In the future, we aim to expand software development facilitating eDQE calculations [18].

Software is freely available via email (magdalena.dobrzynska@ncbj.gov.pl).

## Data Availability

Not applicable.

## Code Availability

Custom code, available freely via corresponding author email.
